# Influence of DOM and its subfractions on the mobilization of heavy metals in rhizosphere soil solution

**DOI:** 10.1038/s41598-022-18419-x

**Published:** 2022-08-18

**Authors:** Meihua Lian, Jun Wang, Yangyang Ma, Jiahui Li, Xiangfeng Zeng

**Affiliations:** 1 Key Laboratory of Wastewater Treatment Technology of Liaoning Province, Shenyang Ligong University, Shenyang, 110159 China; 2 Endocrine Toxicology Branch, Toxicity Assessment Division, National Health and Environmental Effects Research Laboratory, Office of Research and Development, U.S. Environmental Protection Agency, Research Triangle Park, NC 27711 USA; 3 Key Laboratory of Pollution Ecology and Environmental Engineering, Institute of Applied Ecology, Chinese Academy of Sciences, Shenyang, 110016 China

**Keywords:** Environmental sciences, Environmental impact

## Abstract

Long-term industrial pollution, wastewater irrigation, and fertilizer application are known factors that can contribute to the contamination of heavy metals (HMs) in agricultural soil. In addition, dissolved organic matter (DOM) plays key roles in the migration and fate of HMs in soil. This study investigated the effects of amending exogenous DOM extracted from chicken manure (DOMc), humus soil (DOMs), rice husk (DOMr), and its sub-fractions on the mobilization and bio-uptake of Cd, Zn, and Pb. The results suggested that the exogenous DOM facilitate the dissolution of HMs in rhizosphere soil, and the maximum solubility of Zn, Cd, and Pb were 1264.5, 121.3, and 215.7 μg L^−1^, respectively. Moreover, the proportion of Zn-DOM and Cd-DOM increased as the DOM concentration increased, and the highest proportions were 97.5% and 86.9%. However, the proportion of Pb-DOM was stable at > 99% in all treatments. In addition, the proportion of hydrophilic acid (Hy) and Pb/Cd in the rhizosphere soil solution were 17.5% and 8.3%, respectively. This finding suggested that the Hy-metals complex has a vital influence on the mobilization of metals, besides its complexation with fulvic acid and humic acid. Furthermore, the elevated DOM addition contributed to an increment of HMs uptake by *Sedum alfredii*, in the following order, DOMc > DOMs > DOMr. This study can provide valuable insights to enhance the development of phytoremediation technologies and farmland manipulation. Since the risk that exogenous DOM would increase the uptake of HMs by crops, it is also needed to evaluate this case from an agricultural management perspective.

## Introduction

Widespread use of chemical fertilizers and wastewater irrigation is affecting agriculture soils due to contamination of HMs (e.g. Cd, Pb, Zn and Ni)^1^. Therefore, Cd and Pb are common non-essential and poisonous elements in contaminated soils, while Zn is an essential plant nutrient which is a trace element in plants^2^. However, excessive Zn concentrations can negatively affect the metabolism of microorganisms and growth of plants. Moreover, the migration and fate of HMs in soil is dependent on their chemical speciation and influenced by various environmental conditions such as soil agrotypes, soil pH, and dissolved organic matter (DOM)^3^. In fact, DOM, as a highly active material in terrestrial and aquatic ecosystems, represents the mobile phase of organic matter^4,5^. Previous studies suggested that DOM is a heterogeneous mixture and metals can complex with the fulvic acid (FA), humic acid (HA), and other hydrophilic acid by their phenolic hydroxyl, carboxyl and carbonyl groups^6,7^.

Regardless of the source, DOM can either enhance or inhibit the mobility of metals in soils, depending on its origin or specific properties^8^. On one hand, the macromolecular weight, complexation and precipitation with heavy metals of the organic matter can suppress the bioavailability of metals. On the other hand, the low molecular weight DOM can dissolve the metals with the formation of soluble metal–organic complexes, enhancing the metal bioavailability in soils^9^. Consequently, the abundance of the active groups and the dominating composition of DOM predominantly modify the combination with trace metals and hence influence their speciation and bioavailability. Previous studies mainly focused on the effect of endogenous DOM on the speciation of HMs in arable soil, but studies about exogenous DOM have been ignored.

In China, farmland manipulation and application of organic manure and crop residue are important means for the recycling and disposal of agriculture wastes. In fact, this practice has been shown to significantly increase the content of soil DOM^10^. Despite the improvement of the instrumental analyses, it is still difficult to assess the exact composition and structure of DOM due to its heterogeneity. Particularly, little is known about the complexation of exogenous DOM sub-fractions with trace metals in agricultural soil.

DOM is also released in the rhizosphere soil with metals during the planting of hyperaccumulator-like crops. Therefore, plants can directly absorb the trace metals and trigger the excretion of organic acids and other compounds. Also, the rhizosphere environment can further influence the performance of contaminants in soil^11,12^. Previously, it was reported that *Sedum alfredii Hance* (*S. alfredii*) has the potential to remediate soils contaminated with Cd/Zn and other heavy metals^13^. However, it is unclear whether the exogenous DOM and its sub-fractions exhibit different environmental influences on the rhizosphere soil and on the HMs uptake by *S. alfredii*. Therefore, this study analyzed three types of DOM from chicken manure, rice husk, and humus soil, which resembled the exogenous and endogenous DOM application in the farmland. The aim of this study was assess the transformation of Cd, Pb, and Zn in rhizosphere soil solutions and the effects of DOM sub-fractions on the mobilization of HMs. The findings from this study can add to the knowledge about the influence of exogenous DOM on HMs fate in soil. Also, this study can provide valuable insights to facilitate the development of phytoremediation technologies and farmland manipulation.

## Materials and methods

### Experimental design

Soil samples were obtained from the surface layer (0–20 cm depth) in Guchengzi, Liaoning (province of China). The collected soil belongs to the order of Alfisol, and its main physicochemical properties are shown in Table 1. After being air-dried, soil samples were sieved with a 2-mm stainless steel sieve and homogenized. The Cd, Pb, and Zn solutions (3CdSO_4_·8H_2_O, Pb(NO_3_)_2_, Zn(NO_3_)_2_·6H_2_O, respectively) were added to soil in an open plastic container, and the deionized distilled water was then added for soil saturation purposes. After, the soil was allowed to dry under ambient conditions for 10 days before again being re-wetted to saturation with deionized distilled water. The soil was then wetted and dried during seven times^14,15^. The final Cd, Pb, Zn concentrations in the soil were 21.8 mg kg^−1^, 305.4 mg kg^−1^, and 244.7 mg kg^−1^, respectively. Three exogenous DOM extracted from humus soil (DOMs), chicken manure (DOMc), and rice hull (DOMr) were used.

**Table 1:** Physicochemical properties of the collected soil.

pH^a^	Organic C	CEC^b^	Total Zn	Total Pb	Total Cd	Particle size distribution^c^		
–	g kg^−1^	cmol kg^−1^	mg kg^−1^	mg kg^−1^	mg kg^−1^	Sand (%)	Silt (%)	Clay (%)
6.84 ± 0.10	25.70 ± 3.08	17.10 ± 1.25	34.47 ± 2.69	17.23 ± 2.17	0.13 ± 0.04	26.42 ± 3.65	39.81 ± 2.81	33.77 ± 3.52

Data were means ± standard deviations of triplicate experiments.

^a^1:2.5 soil/water ratio.

^b^Cation exchange capacity.

^c^Sand (2–0.02 mm), Silt (0.02–0.002 mm), and Clay (≤ 0.002 mm).

### Pot experiment

Each subsample of 1.0 kg contaminated soil was placed in plastic pots and amended with four levels of each DOM type at 25, 50, 100, and 200 mgC kg^−1^ soil (denoted as T25, T50, T100, T200, respectively). Sufficient amounts of NH_4_NO_3_ were used as a supplement to balance the supply of N. The hyperaccumulating ecotypes of *S. alfredii* of similar sizes were then transplanted into the pots and each treatment was done in quadruplicate. Pot soils with no DOM addition were included as a control (T0). The deionized water was added to keep the water holding capacity approximately at a maximum of 65% by watering every 3–7 days^16^. The plants grew in a greenhouse with controlled light and temperature conditions (20–26 °C day/night temperature)^16^, and were harvested 90 days after the planting.

At the end of the experiment, each plant was separated into root and shoot and oven dried for 48 h at 65 °C. Finally, the rhizosphere soil (i.e. soil tightly adhering to the roots) were collected. The trace metals in plant samples were determined by atomic absorption spectrometry (AAS) after digestion with a mixture of 6 mL HNO_3_ and 4 mL HClO_4_^14^. A certified soil reference material (GBW07401) and blank treatments were used for quality control.

### DOM extraction and fractionation

First, the selected materials of rice hull and chicken manure were air-dried and ground. Samples, including the soil, were mixed with deionized-distilled water with a ratio of 1:20 (w/v) and kept at 25 ± 1 °C for fermentation purposes for a month. Then, the mixture was shaken (200 rpm) for 24 h at 25 °C and centrifuged at 10,000 × g for 20 min^8^. Subsequently, the supernatant was filtered through 0.45 μm microfiltration membranes, and the exogenous DOM was obtained.

DOM was fractionated into three sub-fractions including fulvic (FA) and humic acid (HA), hydrophilic acid (Hy), and hydrophobic neutral organic matter (HON)^17^. At the beginning of experiment, about 25 mL of the sample solution was adjusted to pH 1 with HCl (6 M) to precipitate the dissolved HA^16^. After, the DOC concentration of the pellet with the precipitated HA was measured by removing the supernatant and re-dissolved in 5 mL of 0.1 M KOH^16^. The XAD-8 resin (Sigma) was previously prepared and washed successively with 0.1 M HCl and NaOH solutions, Soxhlet extractions with methanol, and ultrapure water. The clean resin (~ 4 g) was added into the remaining supernatant (representing the dissolved FA + Hy + HON) to adsorb the FA and HON fractions. The suspension was then filtered through a 0.45 μm filter after 1 h of settling by continuous shaking, and a subsample of the filtrate was taken to measure the DOC in the Hy fraction. Additionally, the resin with 10 mL of 0.1 M KOH was equilibrated for 1 h to desorb FA, and the suspension was taken for DOC analysis. Finally, the HON fraction on the resin was quantified as the difference between the total amount of the adsorbed (FA + HON) and the amount of desorbed FA^16,18^. During the experiment, the control was also used to examine the DOC contribution from DAX-8 resin. All the subsamples were analyzed using a total organic carbon (TOC) analyzer (Analytik Jena Multi N/C 3100).

### Soil solution analysis

Each pot was saturated with Milli-Q water and equilibrated for 18 h prior to plant harvesting. Then, the soaking soil was packed into 25-mL filtration tubes and centrifuged at 8000 g for 30 min. The filtration tube was put in a 50-mL centrifuge tube which contained a small spacer in the bottom. The extracted solution was centrifuged (12,000 g, 30 min) again and passed through a 0.45 μm membrane filter. The total dissolved K, Ca, Na, Mg, and anions (SO_4_^2−^, NO_3_^−^, F^−^, Cl^−^, NO_2_^−^) were analyzed by ion chromatography (ICS5000, DIONEX). Total dissolved Cd, Pb, and Zn were quantified by a graphite furnace atomic absorption spectrometry (GFAAS, Varian SpectrAA 220). DOC was analyzed using a TOC analyzer (Analytik Jena Multi N/C 3100).

### Speciation of heavy metals in soil solution

The speciation of Cd, Pb, and Zn in soil solutions was calculated with the Visual MINTEQ software (Version 3.0). Generic parameters, including pH and the concentrations of total DOM (assuming the ratio of DOM to DOC is 2^13^), main anions (SO_4_^2−^, NO_3_^−^, F^−^, Cl^−^, NO_2_^−^), and total dissolved K, Ca, Na, Mg, Cd, Pb, and Zn in soil solution were evaluated^18^. The NICA-Donnan model in Visual MINTEQ was selected to evaluate the metal binding to DOM and calculated using the proportions of HA and FA in the three exogenous DOM according to a previous study^19^.

The exogenous DOMs and the T100 treatment (100 mgC kg^−1^ soil) were considered to further evaluate the influence of DOM sub-fractions on the complexation with metals. Also, the sub-fraction of hydrophilic acid, along with the measured FA and HA fractions, were used to calculate the Cd, Pb, and Zn binding to organic matter. Since the low molecular weight organic acid (LMWOA) is an important constituent of the hydrophilic acid and apt to complex with heavy metals, its parameters were considered in the NICA-Donnan model. Moreover, the LMWOA can account for 30% of hydrophilic acid, including 70% of single carboxylic acid, 20% of dicarboxylic acid, and 10% of tricarboxylic acid (represented as acetic acid, oxalic acid and citric acid, respectively)^20,21^.

### Statistical analysis

Statistical analyses were performed in SPSS 10.0 (IBM Corp.Armonk, NY). Means of different groups were compared using one-way ANOVA. Bar plots and the correlation diagram were plotted by Origin 2022 (Origin Lab, Northampton, MA).

### Statement

The obtained *S. alfredii* was collected from Zhejiang University of China, and the experiments comply with the relevant institutional, national, and international guidelines/legislation in the collection and research on plants.

## Results and discussion

### Concentration of major ions in the soil solution

**Figure 1:**
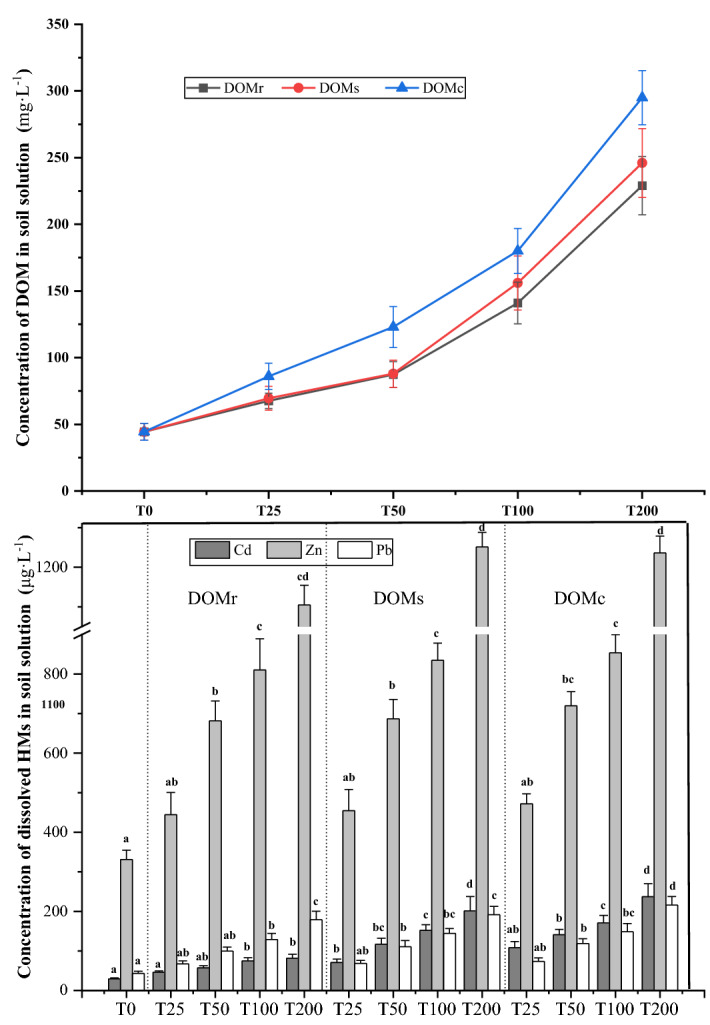
Concentrations of HMs (μg L^−1^) and DOC (mg L^−1^) in soil solution. The lowercase letters refer to significant difference among the treatments for the same HM at 0.05 level. T0, T25, T50, T100, T200 represented for the control, and the treatment of amending with four levels of each DOM at 25, 50, 100, 200 mgC kg^−1^ soil, respectively.

The pH and concentration of the total dissolved Na, K, Mg, Ca, major anions (eg, Cl^−^, NO_2_^−^, SO_4_^2−^, NO_3_^−^, F^−^) in soil solutions are listed in Table S1. The concentration of DOC in the solution increased with the DOM concentration (T200 > T100 > T50 > T25 > T0) (Fig. 1). In fact, the maximum DOM concentrations were 254.2 mg L^−1^, 261.9 mg L^−1^, and 284.2 mg L^−1^ for DOMr, DOMs, and DOMc, respectively (Fig. 1). Overall, the DOM concentration in the rhizosphere soil solution was higher than that of bulk soil with the same treatment, which could be attributed to the root excretion and microbial activities in the rhizosphere area. Moreover, the addition of DOMc resulted in the highest amount of DOC and dissolved Cd, Pb, and Zn, whereas the addition of DOMr yielded a lower amount compared to the other two exogenous DOM. Furthermore, the total dissolved heavy metals raised along with the increasing DOM amount. The concentrations of Cd, Pb, and Zn in the rhizosphere soil solution were 42.5–121.3 μg L^−1^, 65.4–215.7 μg L^−1^ and 446.7–1264.5 μg L^−1^, respectively (Fig. 1).

These findings confirmed that the abundance of active functional groups and structural heterogeneity of DOM can influence the dissolution of heavy metals in the soil solution. In fact, it was reported before that DOMc contains strong active functional groups such as –COOH, C=C, and C=O, which could act as potential chelating centers and facilitate the solubility of metals^19^. Also, Kim et al.^22^ showed that the addition of DOM to soil can lead to decreased S/L (soil solid phase/liquid phase) ratios, desorb metals from soil surface, and promote the dissolution of metals in soil solution. Moreover, the present study showed that the dissolved metals in soil solution increased with the DOM increasing amounts, which agreed with previous studies. Other studies also found that the increasing concentrations of DOC after dairy-manure amendment triggered the dissolution of metals, and there was a further significant positive correlation between the concentration of metals and DOC in the soil solution^5,23^. In this study, the content of Cd and Zn in soil solution was higher than Pb. This finding was consistent with the pK_hydrolysis_ values of Pb^2+^, Cd^2+^ and Zn^2+^ (7.7, 10.08 and 8.96, respectively) because the small hydrolysis constant was usually accompanied with the lower solubility and bioavailability of metals^24^. In addition, the lower solubility of Pb could also be caused by its strong affinity to soil carbonate or Fe/Mn minerals that could limit the release from the solid phase^25^. Moreover, the dynamic equilibrium between the mobilization and replenishment of heavy metals in soil and the uptake by plants could explain the difference of metal concentrations of the rhizosphere and bulk solutions^26^. In this study, the reduction of metals in the rhizosphere soil solution could be related to the higher efficiency of root absorption than that in the replenishment of dissolved metals.

### Speciation of heavy metals in the rhizosphere soil solution

**Figure 2:**
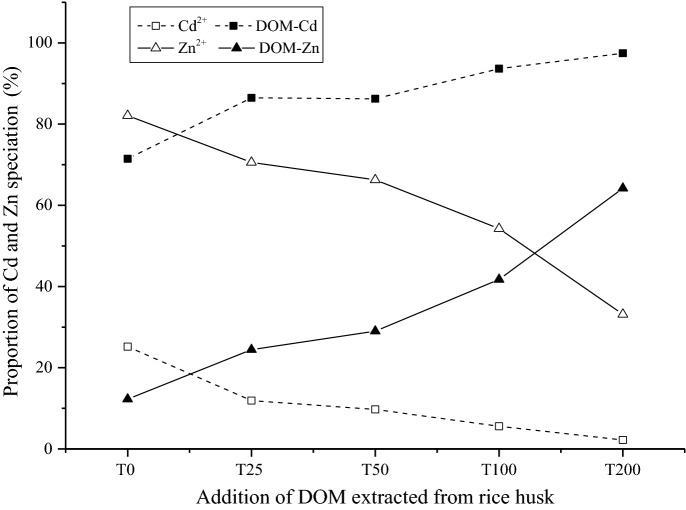
Proportion of Cd and Zn speciation in rhizosphere soil solution of *S. alfredii.*

The speciation distributions of Cd and Zn in the soil solution were calculated by the Visual MINTEQ 3.0 model (Fig. 2 and Figs. S1–S2). Overall, DOMr (Fig. 2) showed the same trend as DOMc and DOMs (Figs. S1 and S2). Additionally, the speciation of metals in the soil solution was determined mainly as free ions and organic complexes. Inorganic complexes, including Metal-Cl^+^, Metal-SO_4_(aq), Metal-NO_3_^+^, Metal-(SO4)_2_^2−^, Metal-NO_2_^+^, only accounted for approximately 2% of the total dissolved metals, and these complexes showed no negligible change under different DOM treatment conditions (Tables S2–S3). Compared to the free ions, the proportions of organic complex Cd and Zn in the rhizosphere soil solution were higher, and these proportions increased along with the DOM addition treatments. Moreover, the highest proportions of organic Cd and Zn complexes in solution were 97.5% (DOMr treatment) and 86.9% (DOMc treatment), respectively. In addition, the proportions of Zn^2+^ in the soil solution were higher than those of Cd^2+^ in the same treatment. For example, the proportion of Zn^2+^ in different treatments ranged from 33.2% (T200) to 70.6% (T25), while Cd^2+^ ranged from 2.2% (T200) to 11.9% in DOMr. In addition, the proportion of free Pb^2+^ was less than 1%, and the organic complex Pb was more than 99% for all treatments (Table S4). There was no inorganic complex Pb species in the soil solution.

These results were consistent with other previous investigations. For instance, Li et al., found that the affinity of Pb with soluble organic ligands in soil solution was significantly stronger than that of Cd and Zn. Therefore, the proportion of organic-Pb was higher^8^. Brümmer investigated the complexation of Cd and Pb with humic acids and demonstrated that the affinity of Pb with humic acids was much higher than that of Cd, with more stable and heterogeneous complexes^27^. The distinct speciation of the three metals in soil solution could be attributed to the differences in chemical characteristics and the ability to form different organic complexes. In addition, Essington indicated that the distribution of electron layers, the ability of accepting electron pairs, and the complexing ability of the same ligand with metal ions is significantly different^24^. In fact, the complexation process of metal ions with organic ligands depends on the stability constant (log*K*_*f*_), with the order of Cu^2+^ > Ni^2+^ > Pb^2+^ > Co^2+^ > Zn^2+^ > Cd^2+^ > Fe^2+^ > Mn^2+^. Therefore, the higher stability constant resulted in more proportion of metal-DOM and accelerated the metal desorbing from the soil solid particles^24,28^.

As concluded from other studies, the NICA-Donnan model can be a reasonable option to predict the metal speciation in the soil solution from wide range of origins, especially for Cd and Zn^18^. However, there is a need to update the generic binding parameters in the model for calculating the Pb speciation. Regarding the Pb speciation, the surface complexation and mineral precipitation/dissolution in the soil should be considered as well. Therefore, the Pb calculation data need a further analysis.

### Effect of the DOM sub-fractions on metal speciation

Sub-fractions of DOM in the soil solution and the proportion of different fractions complexation with metals are shown in Tables 2 and 3. The concentration of DOMs (T100) was 119.4 mg L^−1^ and 149.5 mg L^−1^ in bulk and rhizosphere soil solution, respectively, and it was mainly composed of Hy and FA fractions in the rhizosphere, accounting for 51.5% and 26.4%, respectively. Moreover, the proportions of HA in the bulk solution and HON in the rhizosphere solution were the lowest, with both proportions less than 10%. The NICA-Donnan model calculation indicated that the proportions of the total organic-Cd complex were 83.2%, 8.5%, and 8.3% for FA-Cd, HA-Cd, and Hy-Cd, respectively, while the HA-Pb was the dominant speciation complex followed by FA-Pb and Hy-Pb. There was no speciation detected in the Hy-Zn complex. Therefore, only the HA and FA data were used in the calculation.

**Table 2:** Subfractions of DOM in the soil solution.

Soil solution	DOMs (mg L^−1^)	%Hy	%FA	%HA	%HON
Bulk soil	119.4	42.3	32.2	9.9	15.6
Rhizosphere soil	149.5	51.5	26.4	12.4	9.7

**Table 3:** Proportion of the subfractions of DOM complexed with Cd and Pb in soil solution (%).

Soil solution	FA-Cd	HA-Cd	Hy-Cd	FA-Pb	HA-Pb	Hy-Pb
Bulk	86.4	7.7	5.9	35.7	50.4	13.9
Rhizosphere	83.2	8.5	8.3	24.9	57.6	17.5

In general, studies of DOM-metal complexes in soil solution and natural water mainly focused on the fulvic and humic acids. However, the other hydrophilic acids accounted for one third of DOM in most cases, and the contribution of its complexation ability to heavy metal ions in rhizosphere soil are seldom reported^29^. The findings of this study were different from other previous reports because the effect of LMWOA and other hydrophilic sub-fractions were negligible in terms of the complexation^17,19^. In fact, this study detected more hydrophilic substances in the rhizosphere soil solution due to the root exudation and the contribution of this fraction, especially LMWOA, to the Cd and Pb complexes. In fact, Tao et al. ^12^ indicated that the oxalic, malic, and tartaric acids were the predominant LMWOA in the rhizosphere soil solution of *S. alfredii*. Moreover, the tartaric acid was identified as the unique root exudate, which was mainly distributed within the 0–6 mm rhizosphere range. In fact, the exudation of tartaric acid of LMWOA was highly efficient in the Cd solubilization due to the formation of soluble Cd-tartrate complexes^11^. Despite the low aromaticity of the hydrophilic part, its concentration must be considered when the concentration of active humus is used as an input in the model calculation. Furthermore, the ability of the active humus to complex with heavy metals still needs further investigation^29,30^.

### Effect of DOM on the bio-uptake of Cd, Pb, and Zn by *S. alfredii*

**Figure 3:**
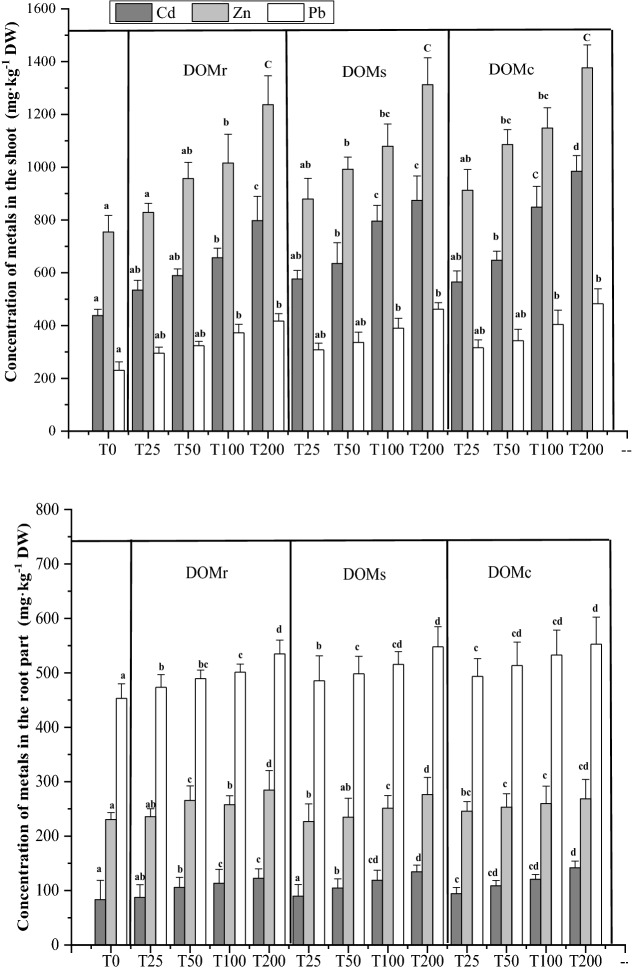
Concentration of Cd, Zn and Pb in the shoot and root of *S. alfredii*. *The lowercase letters refer to significant difference among the treatments for the same HM at 0.05 level.

*Sedum alfredii* was once discovered in an old Pb/ Zn mining area of China and identified as a Zn/Cd co-hyperaccumulator and Pb accumulator from the *Crassulaceae* family^13^. The concentrations of Cd, Pb and Zn in the shoot and root of *S. alfredii* are shown in Fig. 3. Results suggested that the concentrations of HMs in the shoot increased significantly with DOM concentrations (except for Zn at T25) following the order of DOMc > DOMs > DOMr. Specifically, the highest amounts of Cd, Zn, and Pb in the shoot of *S. alfredii* were 984.7 mg kg^−1^, 1376.5 mg kg^−1^, and 482.5 mg kg^−1^, respectively. In addition, concentrations of Cd and Zn in the shoot of *S. alfredii* were higher than those in the root, while the concentration of Pb was lower in the shoot than in the root. Yang et al.^13^ reported that when Pb enters the plant root, it has an intercellular neutral pH, high phosphate, and high carbonate environment, so the transport of Pb from root to shoot is usually low. However, the maximum Pb content in the shoot and root of *S. alfredii* can reach to 514 and 13,922 mg kg^−1^, respectively, when the Pb level was 320 mg L^−1^ ^31^. Furthermore, it was reported that the shoot of *S. alfredii* could uptake up to 5000 mg kg^−1^ Zn and 1400 mg kg^−1^ Cd at pot or field scale^13,32^. Also, the remediating efficiency of *S. alfredii* can vary under different circumstances^33^.

**Figure 4:**
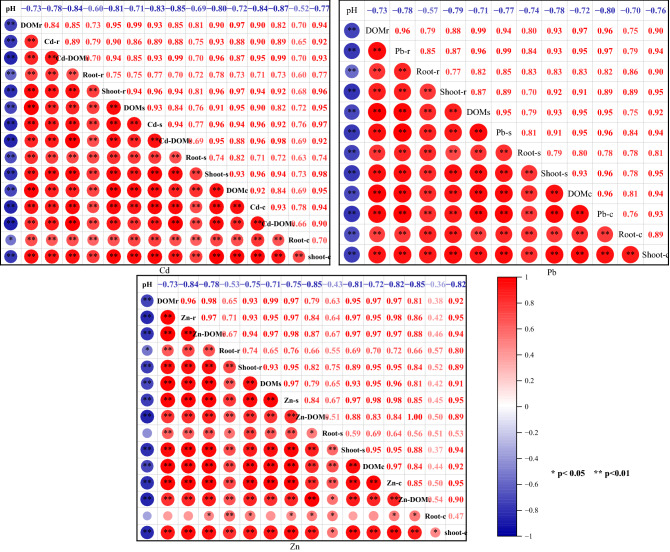
The pearson correlation analysis among pH, DOM concentration, total dissolved HMs, percent of the complex of HMs-DOM and uptake content by the shoot or root of *S. alfredii*. *The lowercase letter of r, s and c stand for the treatment of DOMr, DOMs and DOMc, respectively, and the total dissolved HMs denoted as Cd-, Zn-, Pb-.

The correlation among pH, different types and concentrations of DOM, dissolved metals in soil solution and the uptake HMs by root/shoot of *S. alfredii* were calculated to determine the influence of the DOM addition on the bioavailability of HMs (Fig. 4). A significant positive correlation was observed between DOM and the dissolved HMs and uptake by *S. alfredii* (*P *< 0.01), indicating that the three exogenous DOM can play a key role in the availability of HMs in soil. These observed correlations can be related to the high DOM concentration which can lead to the formation of HMs-DOM complexes and influence the transport and bioavailability of metals afterwards^34^. These findings also revealed that the correlation coefficient of DOM with the Cd and Zn concentrations in the root of *S. alfredii* was weaker than that of Pb, which agreed with the aforementioned results and discussion. Therefore, Cd and Zn could be accumulated in the shoot of *S. alfredii,* while the transfer ability of Pb from root to shoot was small. However, it was not possible to conduct an analysis of the correlation of the HMs speciation and bioavailability due to the limitation of the model in calculating the complexation of Pb-DOM, which needs a further study. Furthermore, although the soil pH did not have an apparent change, a significant negative correlation was observed between pH and the other factors (*P *< 0.05), suggesting that the increased pH was not conductive to the solubility and bioavailability of HMs.

The uptake of heavy metals by plants is influenced by various factors, such as the ecotype of the plant, speciation of metals, and the physicochemical properties of soil. In fact, Krishnamurti and Naidu proposed that the increase of organic matter content in soil after sludge application enhanced the proportion of Cd-DOM complex and the bioavailability of Cd^35^. This enhancement can further promoted the uptake of Cd by plants, which agree with the results of the present study. Moreover, Mao et al. denoted that the FA and some of the HA from compost played regulatory roles in enhancing lateral roots and root hairs and tips, which ameliorated the plant growth and constantly activated the Cd uptake by *S. alfredii* (285.09–709.72 µg plant^−1^)^36^. Also, Fitz and Wenzel demonstrated that hyperaccumulators can enhance the solubility of metals in the rhizosphere soil by root exudation or microbial activity, further promoting the absorption of metals by plants^37^. For example, the intake of exogenous DOM promoted the microbial diversity and community and increased the bioavailability of metals in soil under wetted conditions^38^. In addition, soil DOM can provide nutrient to the microorganisms, and the DOC concentration was positively correlated with the microbial activity. It is well known that soil microbes can affect the mobility and bioavailability of heavy metals by solubilizing metal-phosphates, releasing chelating agents, originating redox changes and through acidification events^39^.

Moreover, the exogenous DOM applied in the HMs-contaminated farmland could mobilize metals which can potentially been taken up by crops or even can be ended up in receiving waters such as rivers and draining streams in agricultural lands. As the uptake and transfer mechanisms of heavy metals by different types of crops are diverse, it is necessary to conduct in-depth research on the absorption of heavy metals and their risk assessment in the future. In addition, the concern of exogenous organic residuals, such as the rise hulls and chicken manure application in agricultural soil, should be addressed.

## Conclusions

In this study, it was demonstrated that the addition of exogenous DOM promoted the dissolution of heavy metals in the rhizosphere soil, and the solubility of Cd and Zn was greater than that of Pb. Also, the proportion of organic complex speciations of Zn and Cd increased significantly with DOM concentrations, and the proportion of Zn^2+^ was higher than Cd^2+^ at the same DOM concentration. Furthermore, the proportion of DOM-Pb in the rhizosphere soil solution was over 99% in all treatments. The complexation of hydrophilic acid with Pb and Cd had an important effect on the mobilization and migration of these two metals, besides the complexation with fulvic acid and humic acid. Furthermore, the significant increasing order of Cd, Pb, and Zn concentrations (except Zn at T_25_) in the shoot of *S. alfredii* were DOM_C_ > DOMs > DOMr. These findings improved the current knowledge about the effects of exogenous DOM on the transformation of trace metals in the rhizosphere soil. Moreover, the application of exogenous DOM could be a useful alternative to improve the efficiency of phytoremediation. However, it is necessary to consider the risk that exogenous DOM can increase the uptake of HMs by crops from an agricultural management perspective.

## Supplementary Information

Supplementary Information.

## Data Availability

The datasets generated during and/or analyzed during the current study are available from the corresponding authors on a reasonable request.
