# Supplementary material for: Influence of DOM and its subfractions on the mobilization of heavy metals in rhizosphere soil solution
Source: Sci Rep. 2022 Aug 18;12:14082. doi: 10.1038/s41598-022-18419-x (PMC9388525; doi:10.1038/s41598-022-18419-x)
Supplement: Supplementary file 1 — Supplementary Information. [file 41598_2022_18419_MOESM1_ESM.docx]

**Title Page**

**Influence of DOM and its subfractions on the** **mobilization of heavy metals in rhizosphere soil solution**

Meihua Lian ^1^, Jun Wang ^2^, Yangyang Ma ^1^, Jiahui Li^1^, Xiangfeng Zeng^3^*

^1^ Key Laboratory of Wastewater Treatment Technology of Liaoning Province, Shenyang Ligong University, Shenyang 110159, China

^2^ Endocrine Toxicology Branch, Toxicity Assessment Division, National Health and Environmental Effects Research Laboratory, Office of Research and Development，U.S. Environmental Protection Agency, Research Triangle Park, North Carolina 27711, United States

^3^ Key Laboratory of Pollution Ecology and Environmental Engineering, Institute of Applied Ecology, Chinese Academy of Sciences, Shenyang 110016, China

* Corresponding author. Email: zengxf@iae.ac.cn

Table S1. Concentrations of the major elements in soil solution

| soil solution | pH | Na | K | Mg | Ca | Cl^-^ | NO_2_^-^ | SO_4_^2-^ | NO_3_^-^ | F^-^ |
| --- | --- | --- | --- | --- | --- | --- | --- | --- | --- | --- |
|  | / | mg· L^-1^ | | | | | | | | |
| Rz ^*^ | 6.46 | 26.32 | 54.16 | 55.37 | 66.31 | 32.86 | 1.24 | 52.39 | 52.0 | 2.66 |
| Bk^**^ | 6.82 | 25.83 | 50.35 | 47.42 | 53.22 | 31.73 | 1.23 | 48.17 | 57.3 | 2.51 |

*rhizosphere soil solution, **bulk soil solution

Table S2. Proportion of the Cd-inorganic in soil solution (%)

| Cd-inorganic | T25 | | T50 | | T100 | | T200 | |
| --- | --- | --- | --- | --- | --- | --- | --- | --- |
|  | Rz | Bk | Rz | Bk | Rz | Bk | Rz | Bk |
| CdF^+^ | 0.02 | 0.025 | 0.017 | 0.022 | - | - | - | - |
| CdCl^+^ | 0.824 | 1.014 | 0.686 | 0.906 | 0.324 | 0.372 | 0.176 | 0.163 |
| CdSO_4_ (aq) | 0.856 | 1.045 | 0.72 | 0.939 | 0.351 | 0.398 | 0.199 | 0.183 |
| CdNO_2_^+^ | 0.021 | 0.026 | 0.017 | 0.023 | - | - | - | - |
| CdNO_3_^+^ | 0.025 | 0.031 | 0.021 | 0.027 | - | 0.011 | - | - |

Table S3. Proportion of the Zn-inorganic in soil solution (%)

| Zn-inorganic | T25 | | T50 | | T100 | | T200 | |
| --- | --- | --- | --- | --- | --- | --- | --- | --- |
|  | Rz | Bk | Rz | Bk | Rz | Bk | Rz | Bk |
| ZnOH^+^ | 0.108 | 0.272 | 0.098 | 0.256 | - | - | - | - |
| Zn(OH)_2_ (aq) | - | 0.024 | - | 0.022 | - | 0.011 | - | - |
| ZnF^+^ | 0.078 | 0.09 | 0.071 | 0.087 | - | 0.051 | - | - |
| ZnCl^+^ | 0.075 | 0.087 | 0.069 | 0.084 | 0.041 | 0.049 | 0.024 | - |
| ZnSO_4_ (aq) | 2.42 | 2.763 | 2.233 | 2.681 | 1.389 | 1.609 | 0.843 | 0.879 |
| Zn(SO_4_)_2_^2-^ | 0.011 | 0.013 | 0.011 | 0.013 | - | - | - | - |
| ZnNO_3_^+^ | 0.06 | 0.069 | 0.055 | 0.066 | 0.033 | 0.039 | 0.019 | 0.02 |

Table S4. Proportion of the speciation of Pb in soil solution (%)

| Pb speciation | T25 | | T50 | | T100 | | T200 | |
| --- | --- | --- | --- | --- | --- | --- | --- | --- |
|  | Rz | Bk | Rz | Bk | Rz | Bk | Rz | Bk |
| Pb^2+^ | 0.067 | 0.059 | 0.057 | 0.056 | 0.027 | 0.022 | 0.015 |  |
| FA-Pb | 59.187 | 66.486 | 59.331 | 66.591 | 57.986 | 64.72 | 55.525 | 63.957 |
| HA-Pb^+^ | 40.717 | 33.427 | 40.586 | 33.327 | 41.967 | 35.242 | 44.442 | 36.023 |

There was no significant difference of the proportion under three DOM treatments, so only the DOMc were listed in the table.

Figure S1. Speciation of Cd and Zn in soil solution of *S. alfredii* after the addition of DOM_S_

Figure S2. Speciation of Cd and Zn in soil solution of *S. alfredii* after the addition of DOM_C_
